# Effectiveness of Multiple Daily Injections or Continuous Subcutaneous Insulin Infusion for Children with Type 1 Diabetes Mellitus in Clinical Practice

**DOI:** 10.1155/2014/526591

**Published:** 2014-08-14

**Authors:** Chun-xiu Gong, Li-ya Wei, Di Wu, Bing-yan Cao, Xi Meng, Lin-lin Wang

**Affiliations:** ^1^Beijing Children's Hospital Affiliated with Capital Medical University, 56 South Lishi Road, Xicheng District, Beijing 100045, China; ^2^Tongzhou Maternal & Child Health Hospital of Beijing, Beijing 101101, China

## Abstract

*Aims*. To determine whether multiple daily injections (MDIs) or continuous subcutaneous insulin infusion (CSII) contributes to better glucose control in children with different type 1 diabetes duration. *Methods*. Subjects were grouped according to early (≤1 year after disease onset; 1A) or late (1–3 years after onset; 2A) MDIs/CSII treatment initiation. Corresponding control groups (1B, 2B) received insulin injections twice daily. *Results*. HbA1c levels were consistently lower in group 1A than in group 1B (6 months (T2): 7.37% versus 8.21%; 12 months (T3): 7.61% versus 8.41%; 24/36 months (T4/T5): 7.61% versus 8.72%; all *P* < 0.05), but were lower in group 2A than in group 2B only at T2 (8.36% versus 9.19%; *P* = 0.04). Levels were lower in group 1A than in group 2A when disease duration was matched (7.61% versus 8.49%; *P* < 0.05). Logistic regression revealed no correlation between HbA1c level and MDIs/CSII therapy. HbA1c levels were only negatively related to insulin dosage. *Conclusions*. Blood glucose control was better in patients receiving MDIs/CSII than in those receiving conventional treatment. Early MDIs/CSII initiation resulted in prolonged maintenance of low HbA1c levels compared with late initiation. MDIs/CSII therapy should be combined with comprehensive management.

## 1. Introduction

Good glycemic control is associated with beneficial outcomes in patients with type 1 diabetes mellitus (T1DM), but it has always been a challenge. The Diabetes Control and Complications Trial (DCCT) and the follow-up Epidemiology of Diabetes Intervention and Complications Study (EDIC) confirmed that intensive insulin treatment could improve glycemic control, reducing or delaying the long-term complications of T1DM, with a persistent benefit [1–3]. Since these results were published, intensive insulin therapy has been widely applied in clinical practice, almost becoming the standard of care [4–6]. However, in contrast to the DCCT and EDIC findings, Holl et al. [7] found that the use of intensive therapy did not improve glucose control in clinical practice in 17 European countries. In addition, a study conducted in the United States showed that glycosylated hemoglobin (HbAlc) levels increased between 2005 and 2011, despite the administration of continuous subcutaneous insulin infusion (CSII) therapy to a greater proportion of patients [8]. Thus, the effects of multiple daily injections (MDIs) and CSII therapies compared with those of conventional therapy remain unclear and require long-term evaluation, especially in clinical practice.

The DCCT [1] provided a strict definition of intensive therapy: MDIs or CSII with blood glucose monitoring more than four times daily. A patient must also adjust his or her insulin dosage according to diet and exercise patterns to maintain the blood glucose level as close to normal as possible. Patients must also be checked monthly at hospitals and frequently communicate with their doctors and nurses by telephone. In actual clinical practice, the appropriate number of injections can be provided easily, but comprehensive management including intensive one-on-one supervision, diet control, psychological counseling, and insulin dosage adjustment is difficult to achieve. Thus, the effects of insulin injection are difficult to distinguish from those of other factors, such as the patient's sex, body mass index (BMI), and diet. Our hospital has implemented intensive therapy and comprehensive management for more than 10 years and applied intensive and conventional therapies to a large number of patients. The present study was conducted to investigate the efficacy of MDIs/CSII in a large sample of Chinese children. We retrospectively analyzed patients with T1DM whom we had followed in long term (2-3 years) to compare MDIs/CSII with conventional treatment and analyze factors associated with HbA1c control.

## 2. Materials and Methods

### 2.1. Research Subjects

Patients with T1DM who were treated at our hospital between 2001 and 2010 were identified using our database of outpatients with diabetes. Children with T1DM who received regular (3-4/year) check-ups at our hospital after diagnosis and for whom complete information about diagnosis and treatment was available were enrolled in this study. Exclusion criteria were as follows: type 2, neonatal, secondary, undetermined, or other types of diabetes; other severe complications; and follow-up period <6 months. Patients who received MDIs or CSII therapy served as experimental subjects. The experimental groups were defined on the basis of the timing of MDIs or CSII therapy initiation: within 1 year of disease onset (group 1A) and 1–3 years after disease onset (group 2A). Because the course of the disease greatly influences blood glucose control, two control groups (1B and 2B) of patients who received insulin injections twice daily were defined for comparison with groups 1A and 2A, respectively. These patients were selected randomly from our database to match the experimental groups in terms of sex, age, disease duration, HbA1c level, insulin dosage (U/kg/d), BMI, and self-monitoring of blood glucose (SMBG).

### 2.2. Study Design and Data Collection

In this study, we retrospectively analyzed treatment effectiveness of MDIs or CSII in comparison with control subjects during a 2-3-year period. The observation time points were baseline (T0) and 3 (T1), 6 (T2), 12 (T3), 24 (T4), and 36 (T5) months. Because follow-up times were gradually reduced with disease duration progression, we selected a date of 24/36 months (T4/T5) as the last observation time point. We collected data on HbA1c level, BMI, insulin dosage, and SMBG frequency from the outpatient database and compared these values among groups and observation time points. In addition, the rate of abnormal glycemic control (HbA1c concentration >9% [9]) was calculated for each of observation time points and compared between experimental and control groups.

### 2.3. Statistical Analysis

Statistical analyses were performed using SPSS software (ver. 17.0; SPSS Inc., Chicago, IL, USA). Means, standard deviations, and proportions were used to describe all study variables. Continuous data were assessed for departures from the normal distribution using the Kolmogorov-Smirnov test. If the data was an approximate normal distribution, parametric tests were utilized; nonparametric tests were applied when distributions did not approximate the normal curve. The comparison between categorical data was done by chi-square test. General linear model (GLM) and generalized estimating equations (GEE) were used to examine the repeated measures data.Univariate and multivariate analyses were performed on blood glucose control (HbA1c <7.5% versus >7.5%). Variables included sex, age, disease duration, follow-up period, number of annual HbA1c tests, BMI (normal versus overweight/obese), insulin dosage, insulin regimen (convention treatment versus MDIs/CSII), and SMBG frequency (<4 versus >4 times/day). Statistically significant variables in univariate analysis were introduced into the logistic regression equation to achieve multivariate analysis. *P* values <0.05 were considered to indicate significant differences.

## 3. Results

At T0, the study sample comprised 252 patients (122 boys and 130 girls). Fifty-eight subjects were lost to follow-up and were excluded from the study. Eight and 14 patients in group 1A dropped out of the study at T3 and T4/T5, respectively. Their age, sex, disease course, HbA1c level, BMI, and insulin dosage did not differ from the remaining patients in group 1A. Three patients in group 2A withdrew from the study; their clinical features did not differ from those of the remaining patients in this group. Thirty-two patients in groups 1B and 2B were excluded from the study; their HbA1c levels did not differ significantly from those of other patients in the control groups.

Group 1A comprised 29 male and 32 female subjects with a mean age of 7.29 ± 3.99 years and a mean disease duration of 1.72 ± 2.43 months (Table 1). Eleven (17%) subjects in group 1A received CSII therapy and 50 (83%) received MDIs. Group 2A comprised 13 male and 10 female subjects with a mean age of 10.50 ± 2.78 years and a mean disease duration of 21.09 ± 5.54 months (Table 1). Five (24%) subjects in group 2A received CSII therapy and 18 (76%) received MDIs.

### 3.1. Baseline Conditions Were Comparable in the Experimental and Control Groups

Groups 1A and 1B did not differ significantly in sex, age, disease duration, HbA1c level, BMI, or SMBG frequency (Table 1). Insulin dosage was significantly lower in group 1B than in group 1A (*P* < 0.05). Groups 2A and 2B did not differ significantly in sex, age, disease duration, HbA1c level, insulin dosage, BMI, or SMBG frequency (Table 1).

### 3.2. MDIs/CSII Treatment Led to Better Glycemic Control than Did Conventional Treatment

**Figure 1:**
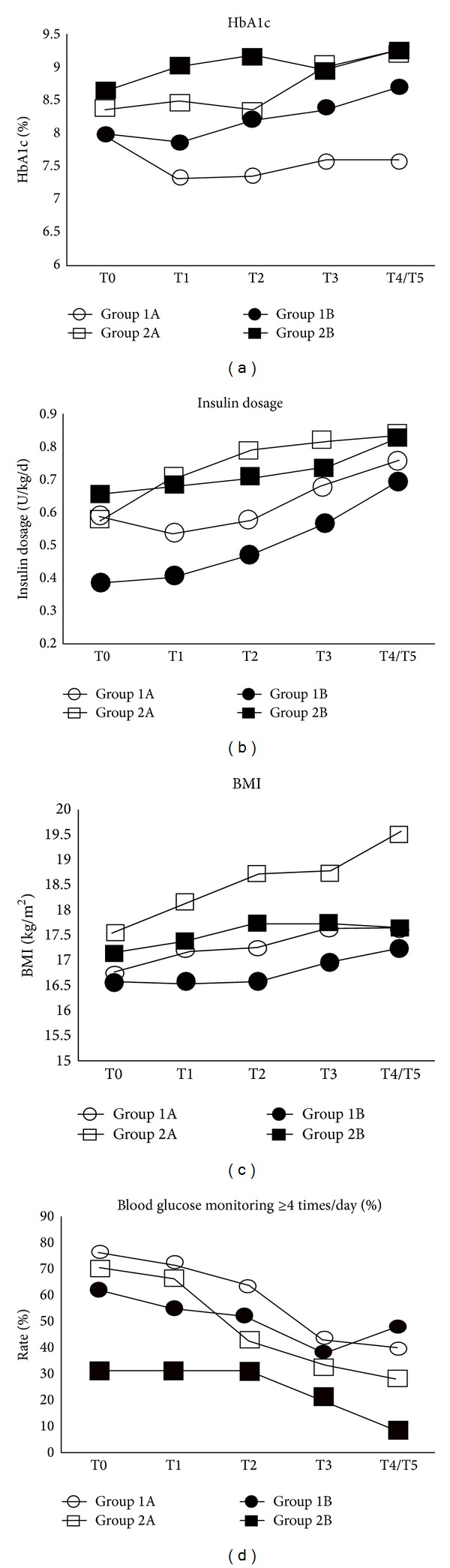
Glycosylated hemoglobin (HbA1c) level (a), insulin dosage (b), body mass index (BMI) (c), and self-monitoring of blood glucose (d) at all observation time points in the control and experimental groups.

HbA1c levels and abnormal control rates were lower in group 1A than in group 1B (both *P* < 0.05). The comparison of groups 1A and 1B at each observation time point is shown in Table 2. Test of within-subjects effects about HbA1c level indicated that it had a tendency to change with time (disease duration) (*F* = 4.054, *P* = 0.005). The HbA1c levels of group 1B were on an upward trend, but it decreased significantly for group 1A at T1 and T2 (*P* < 0.05; Figure 1(a), Table 2). After 12 months of treatment (T3), the HbA1c level of group 1A began to rise and no longer differed significantly from the baseline value (Figure 1(a), Table 2).

The Hb1Ac level and abnormal control rate were lower in group 2A than in group 2B only at 6 months (T2; both *P* < 0.05; Figure 1(a), Table 2). In group 2A, HbA1c levels did not differ significantly from T0 to T4/T5 (Figure 1(a), Table 2). But HbA1c levels of group 2B increased significantly at T2 and T4/T5 (*P* < 0.05; Figure 1(a), Table 2).

HbA1c levels were always lower in group 1A than in group 2A (*P* < 0.05), except at baseline (Figure 1(a), Table 2). Comparison of observation time points matched according to disease duration (T4/T5 in group 1A, T1 in group 2A) showed that the HbA1c level in group 1A was lower than the average level in group 2A (*P* < 0.05). The HbA1c level in group 1A was also lower than the lowest level (T2) in group 2A (*P* < 0.05). The abnormal control rate in group 1A at T4/T5 (15.4%) was lower than those in group 2A at T1 and T2 (26.1% and 21.7%; Table 2).

### 3.3. Insulin Dosage Was Higher in the Early MDIs/CSII Group than in the Control Group

The insulin dosage of four groups increased with disease duration (*P* < 0.05). It was higher in group 1A than in group 1B at T1–T4/T5 (*P* < 0.05; Figure 1(b), Table 2). The insulin dosage did not differ between groups 2A and 2B at any time point (Figure 1(b), Table 2). Comparison of insulin dosage according to matched disease duration (T4/T5 in group 1A, T1 and T2 in group 2A) revealed no significant difference between experimental groups (Figure 1(b), Table 2).

### 3.4. Changes in the BMI Differed between Control and MDIs/CSII Groups

BMI was higher in group 1A than in group 1B at T1 to T3 (*P* < 0.05; Figure 1(c), Table 2). BMI was also higher in group 2A than in group 2B at T1 to T3 (*P* < 0.05; Figure 1(c), Table 2).

### 3.5. SMBG Frequency Did Not Differ between the MDIs/CSII and Control Groups

In group 1A, SMBG frequency had not changed from baseline at T1 or T2 but had clearly decreased at T3 and T4/T5 (*P* < 0.05). No significant difference in SMBG frequency was observed between groups 1A and 1B (Figure 1(d), Table 2).

In group 2A, SMBG frequency had not changed from baseline at T1–T3 but had clearly decreased at T4/T5 (*P* < 0.05). No significant difference in SMBG frequency was observed between groups 2A and 2B (Figure 1(d), Table 2). Comparison of matched disease duration revealed no difference in SMBG frequency between groups 1A (T4/T5) and 2A (T1/T2; Figure 1(d), Table 2).

### 3.6. Correlation Analyses

In the univariate analysis, there was no correlation between HbA1c level and the follow-up period, number of annual HbA1c tests, insulin dosage, insulin regimen, and BMI (*P* > 0.05). The rest of variables were introduced into the logistic regression equation. The results showed that only insulin dosage was associated with glycemic control; that was to say, high insulin dosage was associated with poor glycemic control (Table 3).

## 4. Discussion

Although MDIs/CSII treatment is used widely, its real effects are controversial. We have used the principle supported by the results of the DCCT to manage patients with T1DM for more than 20 years. The present study is the first to document the effects of MDIs/CSII therapy in a large sample of Chinese children with T1DM.

The lower HbA1c levels and lower poor control rates observed in group 1A compared with group 1B indicate that early initiation of MDIs/CSII treatment can result in the maintenance of good glycemic control for 2-3 years. These results are consistent with those of Beck et al. [10], who observed good glycemic control over an 18-month period in patients with T1DM receiving MDIs/CSII therapy. We found a higher baseline insulin dosage in group 1A, indicating that these subjects achieved good glycemic control by having a more positive attitude toward adjustments in insulin dosage. These observations suggest that good glycemic control does not depend solely on MDIs/CSII therapy, but it is the result of active and comprehensive management.

We found that MDIs/CSII treatment suppressed an increase in HbA1c levels for the first 6 months in patients in group 2A. After 6 months, however, HbA1c levels did not differ between groups 2A and 2B. We also found that SMBG frequency decreased with disease progression in these two groups. In other words, SMBG frequency did not increase with the number of injections among patients in group 2A. The refusal of adolescent patients to increase monitoring frequency, as well as their reluctance to follow a strict diet and exercise more frequently, is commonly encountered in clinical practice [4, 7, 11, 12]. We thus believe that the lack of good disease management caused poor glycemic control after 6 months of MDIs/CSII therapy.

Comparison of matched disease duration in groups 1A and 2A revealed lower Hb1Ac levels in group 1A. Patients who converted to MDIs/CSII within 1 year of disease onset clearly had a stronger desire to achieve better blood glucose control than did patients in group 2A and thus complied more with the requirements of comprehensive management. Some studies have shown that the HbA1c level is not related to the number of insulin injections [13]. We believe that the results of our study are in agreement with the finding that compliance with and desire for better glycemic control are more important than the insulin delivery method [14], as they strengthen patients' self-management abilities. Because our study was retrospective and no information about diet and exercise was available for our outpatients, we could not evaluate their comprehensive management abilities.

Taken together, the findings of this 2-3-year follow-up study about Chinese children with T1DM indicate that MDIs/CSII treatment leads to lower HbA1c levels than does conventional treatment and that subjects who started such treatment early showed better glycemic control than did those with late treatment initiation. Based on our results, we recommend the initiation of MDIs/CSII therapy as early as possible but it must be combined with comprehensive management.

## Figures and Tables

**Table 1:** General clinical data at baseline.

	Group 1		P*	Group 2		*P* ^†^
	Group 1A	Group 1B		Group 2A	Group 2B	
Males/females (*n*)	29/32	56/66	0.40	13/10	24/22	0.73
Age (years)	7.29 ± 3.99	8.61 ± 3.08	0.14	10.50 ± 2.78	10.35 ± 2.73	0.84
Disease duration (months)	1.72 ± 2.43	1.77 ± 2.42	0.90	21.09 ± 5.54	21.00 ± 5.72	0.95
Glycosylated hemoglobin level (%)	8.03 ± 1.41	8.01 ± 1.92	0.93	8.39 ± 1.23	8.67 ± 1.44	0.44
Abnormal glycemic control rate (%)	18.0	22.1	0.52	30.4	45.7	0.23
BMI (kg/m^2^)	16.74 ± 2.26	16.57 ± 1.92	0.61	17.54 ± 1.97	17.12 ± 2.45	0.48
Insulin dosage (U/kg/d)	0.59 ± 0.36	0.39 ± 0.24	<0.01	0.58 ± 0.27	0.66 ± 0.23	0.19
Blood glucose monitoring ≥4 times/day (%)	76.3	62	0.15	70	31.3	0.13

∗Group 1A versus group 1B; ^†^group 2A versus group 2B.

**Table 2:** Glycosylated hemoglobin (HbA1c) levels, poor glycemic control rates, insulin dosages, BMI, and frequency of blood glucose monitoring in the experimental (1A, 2A) and control (1B, 2B) groups.

	T0	T1	T2	T3	T4/T5
HbA1c level (%)					
Group 1A	8.03 ± 1.41	7.35 ± 1.24^△^	7.37 ± 1.00^△^	7.61 ± 1.22	7.61 ± 1.15
Group 1B	8.01 ± 1.92	7.89 ± 2.05	8.21 ± 2.05	8.41 ± 2.04	8.72 ± 1.81^△^
P*	0.40	0.10	<0.01	<0.01	<0.01
Group 2A	8.39 ± 1.23	8.49 ± 1.40	8.36 ± 1.25	9.03 ± 1.59	9.24 ± 1.43
Group 2B	8.67 ± 1.44	9.04 ± 1.96	9.19 ± 1.79^△^	8.96 ± 1.94	9.28 ± 1.75^△^
* P* ^†^	0.87	0.48	0.04	0.92	0.40
Poor glycemic control rate (%)					
Group 1A	18	11.5	4.9	13.2	15.4
Group 1B	22.1	24	32.8	28.6	32.7
P*	0.52	0.04	<0.01	0.03	0.04
Group 2A	30.4	26.1	21.7	47.8	45
Group 2B	45.7	43.5	50	37.8	54.1
* P* ^†^	0.23	0.16	0.02	0.43	0.51
Insulin dosage (U/kg/d)					
Group 1A	0.59 ± 0.36	0.54 ± 0.29	0.58 ± 0.26	0.68 ± 0.17	0.76 ± 0.19^△^
Group 1B	0.39 ± 0.24	0.41 ± 0.23	0.47 ± 0.22^△^	0.57 ± 0.23^△^	0.70 ± 0.25^△^
P*	<0.01	<0.01	<0.01	<0.01	<0.01
Group 2A	0.58 ± 0.27	0.72 ± 0.27	0.79 ± 0.25^△^	0.82 ± 0.28^△^	0.84 ± 0.16^△^
Group 2B	0.66 ± 0.23	0.69 ± 0.23	0.71 ± 0.21^△^	0.74 ± 0.20^△^	0.83 ± 0.24^△^
* P* ^†^	0.19	0.42	0.24	0.16	0.67
BMI (kg/m^2^)					
Group 1A	16.74 ± 2.26	17.17 ± 2.00^△^	17.25 ± 2.01	17.61 ± 1.22^△^	17.61 ± 1.15
Group 1B	16.57 ± 1.92	16.57 ± 2.08	16.60 ± 2.07	16.96 ± 2.07^△^	17.21 ± 2.49^△^
P*	0.61	0.02	0.02	0.03	0.53
Group 2A	17.54 ± 1.97	18.13 ± 1.99^△^	18.70 ± 2.67^△^	18.75 ± 2.34^△^	19.50 ± 3.08^△^
Group 2B	17.12 ± 2.45	17.39 ± 2.54	17.73 ± 2.41	17.73 ± 2.57	17.62 ± 2.59^△^
* P* ^†^	0.48	0.07	<0.01	0.03	0.05
Blood glucose monitoring ≥4 times/day (%)					
Group 1A	76.30	71.80	63.40	43.20	40.00
Group 1B	62.00	54.00	51.00	38.50	47.90
P*	0.05	0.09	0.24	0.65	0.49
Group 2A	70.00	66.70	42.90	33.30	28.60
Group 2B	31.30	31.30	30.80	21.40	8.30
* P* ^†^	0.13	0.06	0.52	0.47	0.18

∗Group 1A versus group 1B; ^†^group 2A versus group 2B; ^△^ *P* < 0.05 versus baseline.

**Table 3:** The result of logistic regression about glycosylated hemoglobin levels in the experimental and control groups.

	*B*	Wald	*P*	Exp(*B*)	95% CI
Age	−0.040	1.299	0.254	0.961	0.897–1.029
Disease duration	−0.010	0.776	0.378	0.990	0.969–1.012
Insulin dosage	−2.433	20.222	0.000	0.088	0.030–0.253
Blood glucose monitoring	0.253	1.201	0.273	1.288	0.819–2.025
Constant	1.263	4.349	0.037	3.537	

## Abbreviations

MDIs: Multiple daily injections
CSII: Continuous subcutaneous insulin infusion
HbA1c: Glycosylated hemoglobin
BMI: Body mass index
T1DM: Type 1 diabetes mellitus
SMBG: Self-monitoring of blood glucose
DCCT: Diabetes Control and Complications Trial
EDIC: Epidemiology of Diabetes Intervention and Complications Study.

## References

[1] Diabetes Control and Complications Trial Research Group (1994). Effect of intensive diabetes treatment on the development and progression of long term complications in adolescents with insulin-dependent diabetes mellitus. Diabetes Control and Complications Trial. *Journal of Pediatrics*.

[2] White NW, Cleary PA, Dahms W (2001). Beneficial effects of intensive therapy of diabetes during adolescence: outcomes after the conclusion of the Diabetes Control and Complications Trial (DCCT). *Journal of Pediatrics*.

[3] American Diabetes Association (2002). Implications of the diabetes control and complications trial. *Diabetes Care*.

[4] Al-Agha A, Ocheltree A, Hakeem A (2011). Metabolic control in children and adolescents with insulin-dependent diabetes mellitus at King Abdul-Aziz university hospital. *Journal of Clinical Research in Pediatric Endocrinology*.

[5] Rewers M, Pihoker C, Donaghue K, Hanas R, Swift P, Klingensmith GJ (2007). Assessment and monitoring of glycemic control in children and adolescents with diabetes. *Pediatric Diabetes*.

[6] Nathan DM, Cleary PA, Backlund JC (2005). Intensive diabetes treatment and cardiovascular disease in patients with type 1 diabetes. *The New England Journal of Medicine*.

[7] Holl RW, Swift PGF, Mortensen HB (2003). Insulin injection regimens and metabolic control in an international survey of adolescents with type 1 diabetes over 3 years: results from the Hvidore study group. *European Journal of Pediatrics*.

[8] Klingensmith G, Pihoker G, DuBose S Longitudinal HbA1c values in children and young adults with type 1 diabetes over the last decade: results from the US T1D Exchange clinic registry.

[9] Hanas R, Donaghue K, Klingensmith G (2011). *Global IDF/ISPAD Guideline for Diabetes in Childhood and Adolescence*.

[10] Beck JK, Lewis TV, Logan KJ, Harrison DL, Gardner AW, Copeland KC (2009). Intensive vs. conventional insulin management initiated at diagnosis in children with diabetes: should payer source influence the choice of therapy?. *Pediatric Diabetes*.

[11] Gong CX, Cao BY, Li YC (2008). Re-evaluation of management of children under age of 18 with type 1 diabetes. *Chinese Journal of Diabetes*.

[12] Gong CX, Ni GC, Liu M (2003). Evaluation of management of 123 cases patients with type 1 diabetes and under age of 18 years. *Chinese Journal of Diabetes*.

[13] Gerstl E-M, Rabl W, Rosenbauer J (2008). Metabolic control as reflectet by HbA1c in children, adolescents and young adults with type-1 diabetes mellitus: combined longitudinal analysis including 27,035 patients from 207 centers in Germany and Austria during the last decade. *European Journal of Pediatrics*.

[14] Batajoo RJ, Messina CR, Wilson TA (2012). Long-term efficacy of insulin pump therapy in children with type 1 diabetes mellitus. *Journal of Clinical Research in Pediatric Endocrinology*.

